# *Brain Sciences* - An Open Access Journal

**DOI:** 10.3390/brainsci1010001

**Published:** 2010-07-15

**Authors:** Germán Barrionuevo

**Affiliations:** Founding Editor-in-Chief, Department of Neuroscience, 467 Crawford Hall, University of Pittsburgh, Pittsburgh, PA 15260, USA; E-Mail: german@pitt.edu

During the first ten years that followed “The Decade of the Brain”, the quest of neuroscience for understanding brain function in health and disease has greatly expanded to include molecular, developmental, cognitive and evolutionary aspects of the nervous system. This increased multidisciplinary effort has been complemented by the spectacular development of highly sophisticated experimental methods. Neuroscientists can now perform studies ranging from molecular and imaging analysis of single pre- and postsynaptic neuronal processes to imaging of neural activity in the whole brain during perceptual and motor behavioral tasks. At the same time, theoretical advances in neuroscience have been aided by the rapid development of mathematical and computational simulations of biologically and functionally realistic single cells and complex neural networks across multiple spatiotemporal scales. Therefore, neuroscientists are more than ever in a position to deliver answers to basic, medical and biotechnological questions related to brain function and dysfunction. 

The speedy growth of the neuroscience has manifested itself in the increasing volume of new data, experimental findings, and experimental approaches thus magnifying the need for fast dissemination routes to the science community. However, with print-based peer-reviewed journals the time from original submission of an article until subscribers can access that article averages about two years. The new online, open-access journal *Brain Sciences* has been launched to provide a rapid publishing forum for original peer-reviewed articles from scientists involved in high-quality empirical and theoretical research in the neurosciences. More specifically, *Brain Sciences* publishes original articles, critical reviews, research notes and short communications in the areas of cognitive neuroscience, developmental neuroscience, molecular and cellular neuroscience, neural engineering, neuroimaging, neurolinguistics, neuropathy, systems neuroscience, and theoretical and computational neuroscience. Although meant to assist the reader in focusing on areas of particular interest, this list of topics should not be seen as an exhaustive. Furthermore, we are open to comments and suggestions with respect to additional topics that should receive particular attention within *Brain Sciences*. There is no restriction on the length of the papers, thus giving authors the opportunity to share the experimental and computational protocols, and the data in as much detail as possible. Electronic files, movie clips and software pertaining to the full details of the experimental procedures, and data analyses will be made available as supplementary material. The editorial board is composed of international experts spanning diverse subdisciplines that reflect the broad scope of the field of neuroscience. Their combined and collective expertise will ensure the highest degree of scientific rigor and review of all published articles. We are fully committed to the success of the journal and hope to make *Brain Sciences* a leading outlet to communicate the emerging and exciting research that is taking place in the field of neuroscience. Key to that success is, of course, its readership and potential authors. Thus, we look forward to receiving your contributions in this exciting time for the field of neuroscience. 

